# Further notes on New Zealand Enantiobuninae (Opiliones, Neopilionidae), with the description of a new genus and two new species

**DOI:** 10.3897/zookeys.263.4158

**Published:** 2013-02-04

**Authors:** Christopher K. Taylor

**Affiliations:** 1 Dept of Environment and Agriculture, Curtin University, GPO Box U1987, Perth, WA 6845, Australia

**Keywords:** Palpatores, Phalangioidea, taxonomy

## Abstract

*Mangatangi parvum* **gen. n. and sp.** and *Forsteropsalis pureroa* **sp. n.** are described from the North Island of New Zealand. *Pantopsalis listeri* (White 1849) and *Pantopsalis cheliferoides* (Colenso 1882) are redescribed and no longer regarded as *nomina dubia*; *Pantopsalis luna* (Forster 1944) is identified as a junior synonym of *Pantopsalis listeri*. A key to *Pantopsalis* species is provided.

## Introduction

The Enantiobuninae (*sensu* Taylor 2011, including the Monoscutidae of Crawford 1992) are the dominant group of long-legged harvestmen (Opiliones: Palpatores) found in New Zealand, with over twenty species currently recognised from there (Taylor 2004, 2008, 2011). New Zealand Enantiobuninae can be divided into two separate groups, probably representing distinct clades (Taylor 2011). The first group, previously recognised as the Monoscutinae (Crawford 1992), is only known from three heavily sclerotised species (Forster 1948, Taylor 2008). The greater majority of species belong to the second group, containing taxa previously assigned to the Megalopsalidinae (Crawford 1992). The New Zealand representatives of this group have long been assigned to two genera, *Pantopsalis* and *Megalopsalis*, until Taylor (2011) demonstrated that the New Zealand *‘Megalopsalis’* species were not closely related to the Australian type species of that genus and transferred the bulk of them to a New Zealand endemic genus *Forsteropsalis* (leaving a single anomalous species, *‘Megalopsalis’ triascuta* Forster 1944, whose affinities remain to be demonstrated).

Both genera are represented in museum collections by material from throughout both of the main islands of New Zealand (personal observations). However, accidents of history have resulted in the fauna of the South Island being more extensively investigated than that of the North Island, with the greater number of described species coming from the former. Only one species of *Pantopsalis* and four species of *Forsteropsalis* have been described to date from the North Island. In examining North Island material held in the collection of Te Papa Tongarewa, Wellington (MONZ), a further species of *Forsteropsalis* was recognised, as well as specimens of the previously inadequately described *Pantopsalis cheliferoides*. Examination of these specimens, as well as of specimens attributed to *Pantopsalis listeri* (White 1849) held in the Muséum national d’Histoire naturelle, Paris (MNHP), allowed these two species to be properly characterised and no longer dismissed as *nomina dubia*.

A third novel species from the North Island is of particular interest as it does not accord with either *Pantopsalis* or *Forsteropsalis*, and may represent an outgroup to both. It is here described as representing a new genus.

## Methods

Specimens were sourced from the collections of Te Papa Tongarewa, Wellington, New Zealand (MONZ) and the Muséum national d’Histoire naturelle, Paris, France (MNHP). Photographs and measurements were taken using a Nikon SMZ1500 stereo microscope and the NIS-Elements D 4.00.03 programme, and a Leica DM2500 compound microscope. Coloration is described as in alcohol. Measurements are given in millimetres.

### Mangatangi gen. n.

urn:lsid:zoobank.org:act:1812C6B3-9428-42CB-8855-3609F113E9D7

http://species-id.net/wiki/Mangatangi

#### Type species.

*Mangatangi parvum* new species.

#### Etymology.

Neuter, named for the type locality of the type species.

#### Diagnosis and description.

As for type and only species.

### Mangatangi parvum sp. n.

urn:lsid:zoobank.org:act:F3982336-8FDC-49EF-B17D-E899B32F312D

http://species-id.net/wiki/Mangatangi_parvum

Figure 1

#### Holotype.

♂. New Zealand, **AK.** Mangatangi, Hunua Ra., 8 Feb–8 Mar 1977, I. Barton, ARA Kauri Seed Project, pit trap.

#### Paratypes.

1 ♀, as for holotype; 1 ♂, Cuvier Is, July, R. Forster.

#### Etymology.

From the Latin *parvus*, small, in reference to its small size compared to other New Zealand Enantiobuninae.

#### Diagnosis.

*Mangatangi parvum* can be distinguished from all other New Zealand Enantiobuninae by the presence of a well developed tooth comb on the pedipalpal tarsal claw. It can be distinguished from *Monoscutum titirangiense*, *Acihasta salebrosa* and *Templar incongruens* by its relatively long legs and unsclerotised dorsum. It differs from *‘Megalopsalis’ triascuta*, all *Pantopsalis* and most *Forsteropsalis* species in the absence of either a mediodistal apophysis or hypersetose region on the pedipalpal patella, from *Pantopsalis* species by its relatively bowed cheliceral fingers, and from *Forsteropsalis* species by the absence of denticles on the medial side of the pedipalp coxa. *Mangatangi parvum* can also be distinguished from all other New Zealand species, so far as is known, by its genital morphology: all other New Zealand species investigated to date have a relatively long glans that is either narrow in lateral view (most species) or possesses a distinct dorsal keel (*Pantopsalis*). The deep and short glans of *Mangatangi parvum* is also distinct from that of Enantiobuninae elsewhere: *Neopantopsalis* species have a very elongate and relatively flat glans, and *Megalopsalis* and *Spinicrus* species have a short but also distally flattened glans. The only other Enantiobuninae in Australasia to possess comparatively deep glans are *Australiscutum* and *Tercentenarium linnaei*; *Mangatangi parvum* differs from *Australiscutum* in possessing relatively long legs and retaining an anterior grill of spines over the spiracle, and from *Tercentenarium linnaei* in lacking a large dorsolateral flange at the junction between shaft and glans. The glans of *Thrasychirus* has never been illustrated in lateral view, but *Mangatangi parvum* is clearly distinguished from that genus by possessing paired bristle groups at the junction between shaft and glans rather than single bristles.

#### Description.

**Figure 1.**
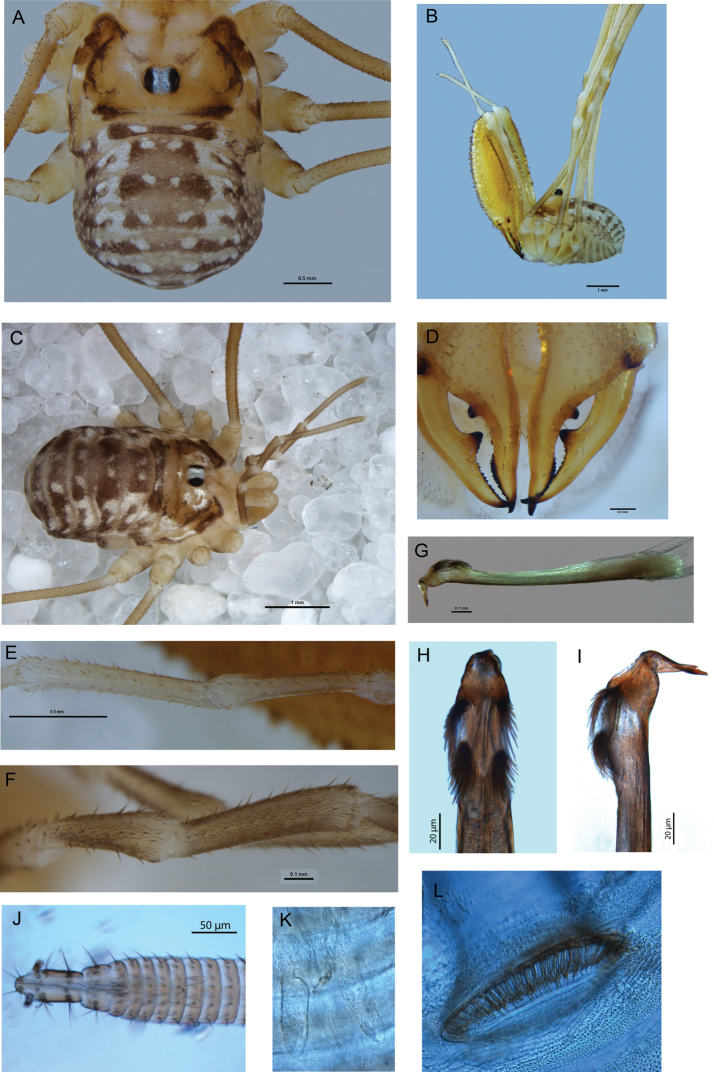
*Mangatangi parvum*. **A** Holotype, dorsal view **B** holotype, lateral view **C** female, dorsal view **D** holotype, cheliceral fingers, anterior view **E** holotype, patella and tibia of left pedipalp, dorsal view **F** female, patella and tibia of right pedipalp, dorsal view **G** penis, right lateral view **H** glans, ventral view **I** glans, left lateral view **J** ovipositor **K** close-up of seminal receptacles **L** left spiracle of female.

*Male* (Figs 1a–b, d–e, g–i, l): Total body length 2.06–2.74 (larger value in all measurements represents holotype), prosoma length 0.97–1.19, prosoma width 1.76–2.01. Dorsal prosomal plate mostly light orange-yellow, unarmed except short, spinose black setae scattered over entire body; anterior propeltidium lighter yellow-cream, supracheliceral groove extending roughly halfway between anterior margin of carapace and ocularium; median propeltidium with diffuse purple stripes along border with anterior propeltidium with diffuse silver-white markings behind purple stripes, dark brown markings on lateral edge of dorsal prosomal plate; ocularium silver with black stripes margining eyes, unarmed; postocularium not distinguished from remainder of posterior propeltidium. Mesopeltidium forming raised ridge, medially pale yellow, laterally dark brown. Ozopores on raised lateral lobes, anterior lobes of prosoma and ozopore lobes dark brown, posterior of ozopore lobes silver-white, remainder of lateral shelves mostly yellow with dark brown lateral margins broadening to diffuse dark brown patch at about three-quarters of distance from front of prosoma. Metapeltidium and dorsum of opisthosoma with background colour of purple broken by pale yellow mottling, particularly along segment boundaries, longitudinal mediolateral broken stripes of silver-white present as well as longitudinal medial rows of silver-white spots, sides of opisthosoma with purple background heavily broken by pale yellow punctations. Mouthparts white; coxae proximally pale yellow; coxae I and II distally with purple mottling, coxae III and IV with dark yellow-brown mottling laterally; genital operculum pale yellow; venter of opisthosoma mottled light purple with pale yellow stripes along segment boundaries.

*Chelicerae*: Segment I 2.85–3.51, segment II 3.82–4.62. Segment I ventrally cream, dorsally orange-yellow, sparsely denticulate dorsally; segment II inflated, orange-yellow, densely dorsally and sparsely ventrally denticulate. Cheliceral fingers (Fig. 1d) long, bowed, movable finger with setae close to median tooth.

*Pedipalps*: Femur 1.53–2.13, patella 0.65–0.77, tibia 0.71–1.03, tarsus 1.80–2.47. Coxae unarmed. Femur to tarsus long, slender, unarmed, femur to tibia cream with paler distal ends to each segment, tarsus off-white with yellow-brown shading at distal end. Patella and tibia (Fig. 1e) straight, patella without distal prolateral apophysis or hypersetose region. Plumose setae absent. Microtrichia on distal half of tarsus only. Claw with ventral tooth-comb.

*Legs*: Leg I femur 2.99–3.80, patella 0.71–0.91, tibia 2.93–3.87; leg II not preserved; leg III 2.56–3.38, patella 0.77–0.93, tibia 2.70–3.45; leg IV femur 4.05–5.01, patella and tibia not preserved. All segments unarmed. Trochanters pale yellow, trochanters III and IV with dark yellow-brown mottling laterally. Femora to tarsi pale yellow, patellae and distal ends of femora and tibiae darkening to orange-yellow. Leg II not preserved; tibia IV with three pseudosegments.

*Penis* (Figs 1g–i): Glans noticeably short and deep, sides parabolic in ventral view. Bristle groups of medium length. Tendon short, not extending far behind bristle groups.

*Spiracle* (Fig. 1l): Curtain of distally anastomosing spines extending over entire spiracle; shortening to cluster of tubercles (possibly lace tubercles) at medial corner.

*Female* (Figs 1c, f, j–k): Coloration similar to that of male. Other features as for male except for following: Chelicerae not enlarged, unarmed, segment I without ventral spine. Pedipalp (Fig. 1f) with microtrichia over entire patella, tibia and tarsus except glabrous dorsal line on patella and tibia. Ovipositor (Figs 1j–k) with single pair of seminal receptacles.

#### Phylogeny.

*Mangatangi parvum* is probably related to the clade formed by *Forsteropsalis* and *Pantopsalis*, with which it shares the presence of sharp papillae on the glans, and of setae close to the major tooth of the mobile finger of the chelicera (this last feature is also present in *Neopantopsalis*). The retention in *Mangatangi parvum* of a plesiomorphic tooth-comb on the pedipalpal tarsal claw, together with *Mangatangi parvum*’s distinctly short glans, could suggest a sister relationship between *Mangatangi parvum* and the *Pantopsalis* + *Forsteropsalis* clade, but this should perhaps be treated with caution. *Pantopsalis rennelli* and *Pantopsalis cheliferoides* each retain reduced teeth arrays (a single tooth in the latter species) on the tarsal claw, and that of *Pantopsalis albipalpis* has a ventral rugose area that may correspond to the remains of the tooth-row. The loss of the tooth-row in *Pantopsalis* and *Forsteropsalis* has therefore happened at least partially in parallel. As regards the short glans of *Mangatangi parvum* compared to the long glans of *Pantopsalis* and *Forsteropsalis*, our understanding of enantiobunine phylogeny is not yet robustly resolved (Taylor 2011) and it is questionable which state is plesiomorphic for the clade.

### Forsteropsalis pureora sp. n.

urn:lsid:zoobank.org:act:F97E7775-669A-42CB-81ED-5578B8997191

http://species-id.net/wiki/Forsteropsalis_pureora

Figure 2

#### Holotype.

♂. New Zealand, **TO.** Pureora, Waipapa Reserve, 570 m, 15 December 1983, J. Hutchinson, malaise trap in shrublands.

#### Etymology.

Named for the type locality.

#### Diagnosis.

The genus *Forsteropsalis* was established and revised by Taylor (2011). In the key to *Forsteropsalis* provided therein, *Forsteropsalis pureora* can easily be taken as far as couplet 6: it differs from *Forsteropsalis grimmetti* in lacking the latter’s flattened and ventrally white opisthosoma, from *Forsteropsalis fabulosa* and *Forsteropsalis tumida* in not having the chelicerae greatly inflated and the cheliceral fingers widely bowed, and from *Forsteropsalis inconstans* and *Forsteropsalis nigra* in not having the posterior part of the propeltidium and the mesopeltidium heavily denticulate. The only species with which *Forsteropsalis pureora* is likely to be confused are *Forsteropsalis distincta*, *Forsteropsalis chiltoni*, *Forsteropsalis marplesi* and *Forsteropsalis wattsi*. *Forsteropsalis pureora* can be distinguished from *Forsteropsalis distincta* by the presence of denticles in the anterior propeltidial area, whereas *Forsteropsalis distincta* has the prosomal dorsum unarmed but for the anterior corners. From *Forsteropsalis chiltoni*, *Forsteropsalis marplesi* and *Forsteropsalis wattsi*, *Forsteropsalis pureora* can be distinguished by the absence of a distinct pedipalpal patellar apophysis. It can also be distinguished from *Forsteropsalis wattsi* by the absence in the latter of denticles on the femora. *Forsteropsalis chiltoni* and *Forsteropsalis marplesi* are larger species (both have the prosoma more than 2 mm in length) with more developed denticulation at the anterior corners of the prosoma, and without any medial stripe on the opisthosoma. The latter two species also differ from *Forsteropsalis pureora* in genital morphology: both have the glans in ventral view narrowing anterior to the lateral bristle groups (Taylor 2011, Figs 97, 118) while that of *Forsteropsalis pureora* is more constant in width.

#### Description.

*Male*: Total body length 3.73, prosoma length 1.77, prosoma width 2.83. Dorsal prosomal plate honey-brown with large white patches covering much of median propeltidium on either side of ocularium and becoming diffuse behind ocularium, postocularial area yellow-grey; anterior propeltidium with sharp denticles, remainder of dorsum unarmed but with scattered black setae; ocularium white. Mesopeltidium forming raised ridge, medially pale yellow-grey, darkening to honey-brown laterally. Ozopores on raised lateral lobes, anterior ozopore lobe with distinct white patch, remainder of lateral shelves honey-brown with smaller white patches on posterior ozopore lobe and near posterior end of shelf. Metapeltidium and dorsum of opisthosoma light purple with median white stripe and transverse rows of white spots across segments. Mouthparts white; coxae mottled honey-brown proximally, darker brown distally; genital operculum honey-brown; venter of opisthosoma light purple.

**Figure 2.**
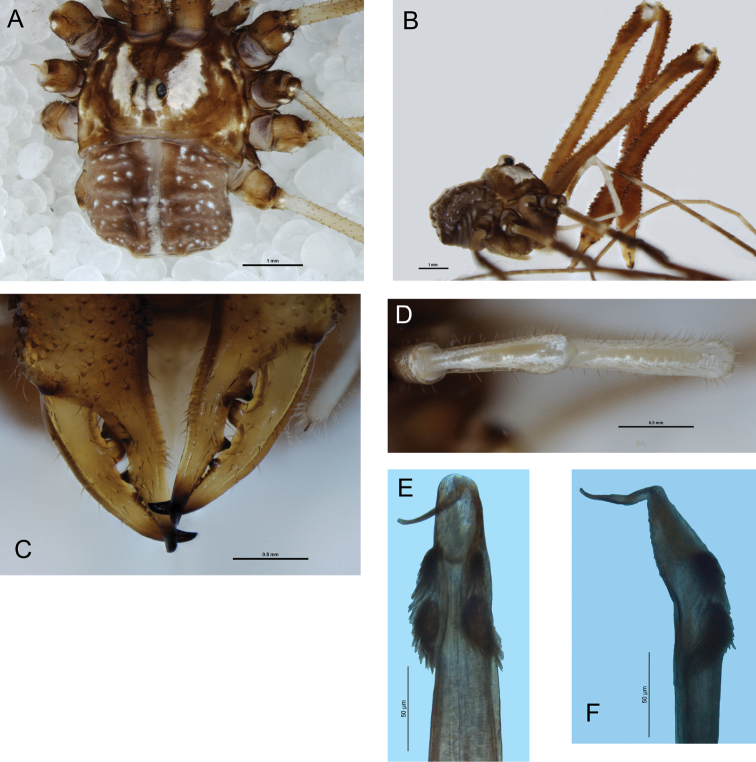
*Forsteropsalis pureroa*, holotype. **A** Dorsal view **B** lateral view **C** cheliceral fingers, anterior view **D** patella and tibia of right pedipalp, dorsal view **E** glans, ventral view **F** glans, right lateral view.

*Chelicerae*: Segment I 7.06, segment II 8.94. Segment I darker yellow-brown with white patches at distal end; segment II orange-brown; both segments evenly denticulate; segment II not inflated. Cheliceral fingers (Fig. 2c) long, only slightly bowed, movable finger with numerous setae close to median tooth.

*Pedipalps*: Femur 2.00, patella 0.98, tibia 1.21, tarsus 2.75. Coxa with numerous sturdy denticles on prolateral margin. Pedipalps long, slender, femur with dorsal and ventral rows of denticles; femur proximally honey-brown except cream-coloured heel; distal end of femur, patella and tibia white mottled with cream; tarsus cream-coloured. Patella and tibia (Fig. 2d) straight, patella with concentration of strong setae at prolateral distal end but without distinct apophysis. Microtrichia on distal two-thirds of tarsus. Tarsal claw ventrally rugose.

*Legs*: Leg I femur 7.03, patella 1.48, tibia 6.04; leg II femur 11.70, patella 1.74, tibia 11.64; leg III femur 7.00, patella 1.51, tibia 5.44; leg IV femur 8.32, patella 1.38, tibia 7.83. Femora with relatively few small denticles dorsally; other segments unarmed. Trochanters honey-brown with white distal retrolateral spot on each trochanter. Femora proximally dull medium yellow, distal ends of femora to tibiae honey-brown mottled with white spots; tibiae lighter orange-brown banded with dull yellow. Tibia I with two pseudosegments; tibia II with ten pseudosegments; tibia IV with three pseudosegments.

*Penis* (Figs 2e–f): Glans relatively long, sides parabolic in ventral view; triangular in lateral view. Bristle groups of medium length. Tendon long.

#### Comments.

Another feature of *Forsteropsalis pureora* that may deserve further investigation is the unusually high number of pseudosegments in the leg tibiae. Not only does the holotype have a higher number of pseudosegments in tibia II than recorded for any other *Forsteropsalis* species, even the particularly large species *Forsteropsalis fabulosa* and *Forsteropsalis tumida*, it represents the first recorded instance in this genus of pseudosegmentation in tibia I. At our present level of knowledge, this cannot be considered a reliable distinguishing character for the species as tibial pseudosegment number has been found in other species to vary between individuals. However, pseudosegment number has been suggested to distinguish *Forsteropsalis chiltoni* and *Forsteropsalis marplesi* in which, so far as is known, males of each species have varying but non-overlapping ranges of pseudosegment number for tibia II (Taylor 2011). This may reflect differences in leg proportions between the two species: despite *Forsteropsalis chiltoni* having a generally larger body size than *Forsteropsalis marplesi* (average prosomal length 3.1 mm in two specimens of *Forsteropsalis chiltoni* vs 2.5 mm in four specimens of *Forsteropsalis marplesi*), *Forsteropsalis marplesi* specimens may have relatively longer legs (average length of tibia II 9.8 mm in *Forsteropsalis chiltoni* vs 11.8 mm in *Forsteropsalis marplesi*) (unpublished personal observations). Like examined specimens of *Forsteropsalis marplesi*, the holotype of *Forsteropsalis pureora* has relatively long legs compared to body size. However, the significance of these observations remains open to question. Previous morphometric studies of other Opiliones have found leg measurements to be useful in distinguishing taxa among Goniosomatinae (Laniatores; Gnaspini 1999) but not *Leiobunum* (McGhee 1977). A detailed morphometric study to establish the reliability and/or significance of such measurements in distinguishing taxa will require a much larger sample of specimens than currently available for most *Forsteropsalis* species.

### Pantopsalis listeri

(White, 1849)

http://species-id.net/wiki/Pantopsalis_listeri

Figure 3

Phalangium listeri White 1849: 6 (reprinted 1850: 52).
Pantopsalis listeri (White 1849): Simon 1879: 73, Taylor 2004: 61 (as *nomen dubium*; further citations provided by Taylor 2004).
Megalopsalis luna Forster 1944: 190, pl. 66 Figs 1–3 **syn. n.**
Pantopsalis luna (Forster 1944): Taylor 2004: 70–71, Fig. 9.

#### Neotype. 

1 ♂, ‘Ile du Milieu, Filhol, 1875-75’ (MNHP no. 134).

#### Other specimens examined.

1 ♂, same data as neotype; 3 ♂, New Zealand, **WD.** Waiho Gorge, Sth Westland, 21 July 1927 (MONZ); photographs of live males provided by Simon Pollard (Canterbury Museum, Christchurch).

#### Description.

**Figure 3.**
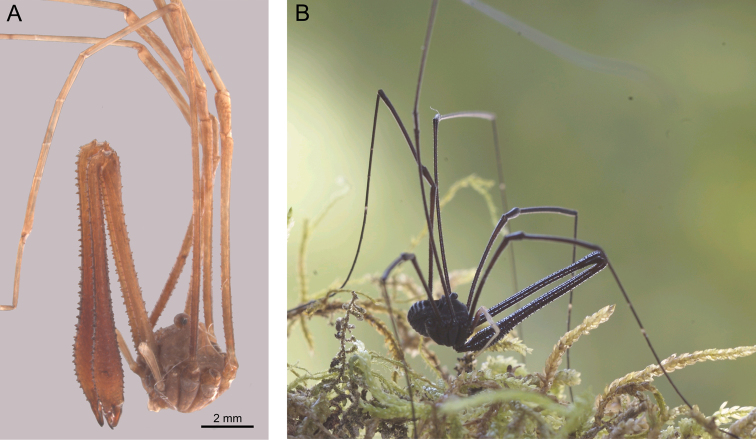
*Pantopsalis listeri*. **A** Neotype, lateral view **B** live specimen, photographed by Simon Pollard.

As described by Simon (1879), with the following additions: Dorsum of opisthosoma with pale silvery, narrow, transverse stripes on posterior margins of segments (Fig. 3b). Segment II of chelicera inflated in neotype, slender in remaining specimens. Glans of penis without dorsal keel (Taylor 2004: Fig. 9).

#### Comments.

The original type specimen(s) of *Phalangium listeri* are lost; they have not been located in the collection of the Museum of Natural History, London (J. Beccaloni, pers. com.) or of the Muséum national d’Histoire naturelle, Paris (M. Judson, pers. com.) It was therefore treated as a *nomen dubium* by Taylor (2004). A redescription of *Pantopsalis listeri* was provided by Simon (1879), who made it the type species of his new genus *Pantopsalis*.

Since the publication of Taylor (2004), I have had the opportunity to examine two of the specimens used by Simon (1879) in his redescription of this species. Their state of preservation is not ideal (they appear to have been subject to desiccation at some point in the past) and the genitalia have become distorted. Nevertheless, I was able to confirm the absence of a dorsal keel on the glans, demonstrating that *Pantopsalis listeri* could not be conspecific with *Pantopsalis albipalpis*, *Pantopsalis coronata* or *Pantopsalis phocator* among other South Island *Pantopsalis* species. External characters (described by Simon 1879), such as the unarmed ocularium with denticles restricted to the anterior propeltidium on the dorsal prosomal plate, are also consistent with male specimens referred to *Pantopsalis luna* (Forster 1944) by Taylor (2004) (pers. obs.), and it is my judgement that that species is a junior synonym of *Pantopsalis listeri* sensu Simon (1879). The opisthosoma has collapsed in both MNHP specimens, so Simon’s (1879) failure to note the transverse striping present in this species may be an artefact of preservation and does not oppose the synonymy.

White’s (1849) original description of *Pantopsalis listeri* does not provide a more detailed type locality than ‘New Zealand’, but Judson (1997) suggested the Bay of Islands, North Island, as the likely type locality for *Chelifer pallipes* White 1849 (now *Philomaoria pallipes*), described in the same paper. If this was also the case for *Pantopsalis listeri*, then Simon’s (1879) specimens would be unlikely to represent the same species as White’s original type(s). Simon (1879) did not explicitly indicate how he identified his specimens as *Pantopsalis listeri*; as no other New Zealand enantiobunine had yet been described, Simon was probably simply unaware that more than one phalangioid species with enlarged chelicerae existed there. Nevertheless, one of the MNHP specimens (Fig. 3a) is here designated as neotype of *Phalangium listeri*. White’s original description was exceedingly rudimentary, giving basic characters of the chelicerae only, and inadequate for determining which of the genera *Pantopsalis*, *Forsteropsalis* or *Mangatangi* was being examined. Simon’s more detailed redescription was at least implicitly used as the basis for identification of *Pantopsalis* by all subsequent authors (Pocock 1903a, b, Hogg 1910, 1920, Roewer 1923, Taylor 2004). Nomenclatural stability is best served by fixing *Pantopsalis listeri*’s identity as the species examined by Simon.

### Pantopsalis cheliferoides

(Colenso, 1882)

http://species-id.net/wiki/Pantopsalis_cheliferoides

Figure 4

Phalangium (Phrynus) cheliferoides Colenso 1882: 166.
Pantopsalis cheliferoides (Colenso 1882): Vink *in*Nicholls et al. 2000: 46, Taylor 2004: 65 (as *nomen dubium*).

#### Specimens examined.

1 ♂, New Zealand, **GB.** Lake Waikaremoana, 19 November 1975, G. W. Ransay, beating; 1 ♂, New Zealand, **GB.** Te Urewera National Park, Lake Waikaremoana, Kaitawa, 38°46'S, 177°83'E, 18 November 2004, D. King, on outside of house.

#### Description:

*Male*: Total body length 3.55–4.03 (former measurement refers to 2004 specimen), prosoma length 1.62–2.06, prosoma width 2.55–3.70. Dorsal prosomal plate medium brown, with some yellowish patches laterally; anterior propeltidium and ocularium heavily denticulate, remainder of prosoma unarmed. Dorsum of opisthosoma dark purplish brown with few white spots medially in one specimen, longitudinal purple medial stripe in other; larger white spots in present in central part of opisthosomal dorsum, comparable to lateral ‘arms’ of median stripe in females of other *Pantopsalis* species. Coxae medium brown mottled with honey brown; venter of opisthosoma medium purplish brown mottled with lighter purple.

**Figure 4.**
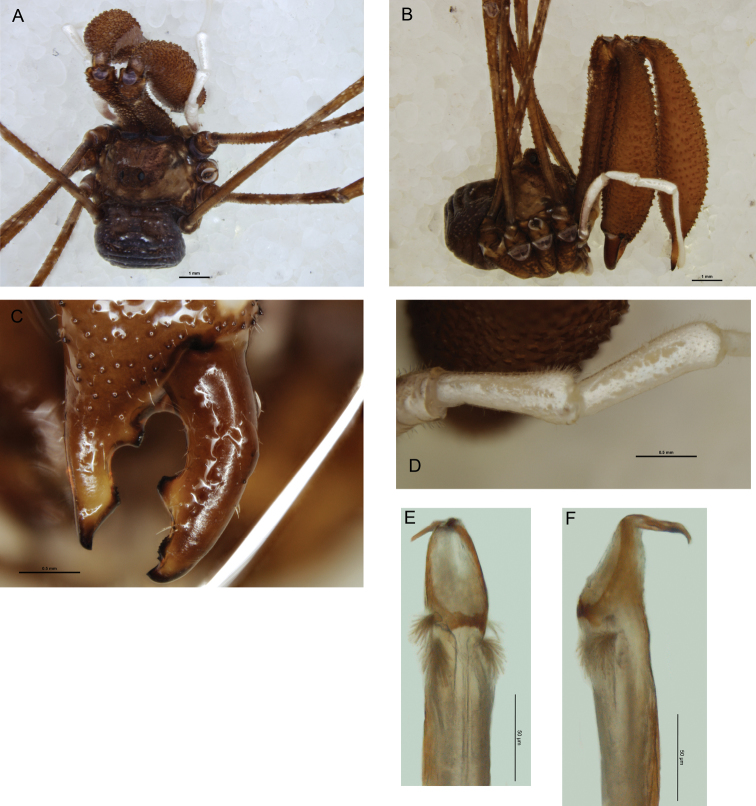
*Pantopsalis cheliferoides*, specimen collected in 1975. **A** Dorsal view **B** lateral view **C** fingers of left chelicera, anterior view **D** patella and tibia of right pedipalp, dorsal view **E** glans, ventral view **F** glans, left lateral view.

*Chelicerae*: Segment I 4.73–6.23, segment II 6.36–8.47. Segment I medium brown with cream patches at distal end; segment II orange-brown; both segments heavily denticulate. Segment II inflated in larger specimen, slender in smaller. Cheliceral fingers (Fig. 4c) short, mobile finger crescent-shaped.

*Pedipalps*: Femur 1.81–2.32, patella 0.97–1.15, tibia 1.01–1.18, tarsus 2.10–3.02. Femur light purple at base, remainder of pedipalp shining white. Patella and tibia (Fig. 4d) prolaterally hypersetose, patella bulging prodistally but without distinct apophysis. Microtrichia on distalmost end of tarsus only. Tarsal claw with single ventral tooth.

*Legs*: Leg I femur 7.91–8.56, patella 1.36–1.58, tibia 6.03–6.14; leg II femur 13.25–13.69, patella 1.82–1.80, tibia 11.18–11.47; leg III femur 6.91–7.38, patella 1.32–1.45, tibia 5.22–5.48; leg IV femur 9.88–10.81, patella 1.57–1.59, tibia7.53–7.38. Femora evenly but irregularly denticulate, except distal third of femur II unarmed; remaining segments unarmed. Legs medium brown mottled with yellowish, tibiae and tarsi tinged with purple, tibiae spotted with white; tarsi with white band at base of telotarsi. Tibia II with five pseudosegments; tibia IV undivided in larger specimen, with two pseudosegments in smaller.

*Penis* (Fig. 4e–f): Glans medium length, sides parabolic in ventral view; subtriangular in lateral view but not markedly flattened, slight dorsomedial bulge but keel essentially absent. Bristle groups short. Tendon long.

#### Comments.

There is some variation in coloration between the two specimens available, most notably the presence of a medial stripe on the opisthosoma of one but not the other, with the former specimen also being overall lighter in coloration than the latter. It is possible that this difference may reflect differences in maturity between the two specimens, similar to what has been recorded for other Opiliones species (Shultz 2008). Such long-term darkening after moulting may also explain the anomalously pale specimens of *Pantopsalis albipalpis* recorded by Taylor (2004).

Long regarded as something of a mystery after its initial description by Colenso (1882), *Pantopsalis cheliferoides* was referred to only in footnotes by Forster (1944) and Marples (1944), and treated as a *nomen dubium* by Taylor (2004) due to the poor condition of the type specimen. This was unfortunate as, with the recognition of *‘Pantopsalis’ wattsi* Hogg 1920 as a species of *Forsteropsalis* (Taylor 2011), *Pantopsalis cheliferoides* was the only species of *Pantopsalis* described from the North Island. The two specimens examined here, though collected some distance from the type locality, are consistent with Colenso’s (1882) original description and discernable features of the type specimen (Taylor 2004) and, in the absence of evidence to the contrary, can be identified as *Pantopsalis cheliferoides*. This species can, therefore, be confirmed as distinct from other identifiable species of *Pantopsalis*.

The absence of a distinct dorsal keel on the glans of the penis clearly distinguishes *Pantopsalis cheliferoides* from all other *Pantopsalis* species except *Pantopsalis luna* and possibly *Pantopsalis pococki* (for which the genital morphology remains unknown). *Pantopsalis pococki* has a very distinct colour pattern, with transverse light coloured stripes on the dorsum of the opisthosoma (Taylor 2004). *Pantopsalis luna* lacks a ventral tooth on the pedipalpal tarsal claw, has the ocularium unarmed with denticulation on the prosoma restricted to the anterior propeltidial region, and has narrow transverse stripes on the dorsum of the opisthosoma (personal observations, male specimens from Waiho Gorge, South Island, cited by Taylor 2004).

The presence of dimorphic males as described for other *Pantopsalis* species by Taylor (2004) in both *Pantopsalis listeri* and *Pantopsalis cheliferoides* is of note. Previous species in which this phenomenon has been observed (*Pantopsalis albipalpis*, *Pantopsalis coronata*, *Pantopsalis johnsi* and *Pantopsalis phocator*) all belong to the well-marked species group whose members possess a strong dorsal keel on the glans (Taylor 2004) and it was previously unknown whether such male dimorphism occurred outside this species group. Its presence in the two species treated herein indicates that it does, and it may indeed be a synapomorphy for *Pantopsalis* as a whole.

##### Key to males of *Pantopsalis*

The last author to provide a key to species of *Pantopsalis* was Roewer (1923). Since then, a number of species have been described, several of the species referred to by Roewer have been synonymised, and characters of the chelicerae referred to by Roewer have been shown to vary within species (Taylor 2004). Therefore, the opportunity is taken to provide an updated key to *Pantopsalis* males. *Pantopsalis halli* Hogg 1920 is omitted from the following key, as it is currently based only on a female specimen and not identifiable (Taylor 2004). *Pantopsalis albipalpis* and *Pantopsalis johnsi* are not currently distinguishable, but Taylor (2004) refrained from synonymising them on the basis of their widely disjunct distributions. Their relationship requires further investigation.

**Table:** 

1	Lateral parts of opisthosoma with extensive light-coloured markings, either broadly light-coloured or with broad transverse stripes, contrasting with darker median; light coloured transverse stripe often covering most of metapeltidium and/or first opisthosomal segment	2
–	Lateral parts of opisthosoma largely dark (longitudinal median stripe may be present; transverse stripes, if present, narrow and not covering most of lateral part of opisthosoma), no light transverse stripe over metapeltidium and first opisthosomal segment	4
2	Light-coloured lateral patches extending mediad as transverse stripes; articular membranes not brightly coloured	3
–	Light-coloured patches restricted to lateral part of opisthosoma, not extending mediad as transverse stripes; articular membranes brightly coloured (white in alcohol)	*Pantopsalis phocator* Taylor 2004
3	Dorsal prosomal plate with numerous well-developed denticles in both anterior and medial propeltidial areas	*Pantopsalis pococki* Hogg 1920
–	Dorsal prosomal plate with few denticles, and those low and rounded	*Pantopsalis coronata* Pocock 1903b
4	Dorsal prosomal plate with denticles in anterior propeltidial area at least	5
–	Dorsal prosomal plate completely unarmed	7
5	Glans of penis with well-developed dorsal keel	*Pantopsalis albipalpis* Pocock 1903a (Otago) or *Pantopsalis johnsi* Forster 1964 (Auckland Islands)
–	Glans of penis without distinct dorsal keel	6
6	Ocularium unarmed; opisthosoma with narrow light-coloured transverse stripes	*Pantopsalis listeri* (White 1849)
–	Ocularium denticulate; opisthosoma without transverse stripes	*Pantopsalis cheliferoides* (Colenso 1882)
7	Length of pedipalp femur less than half width of prosoma; femora of legs with few denticles	*Pantopsalis rennelli* Forster 1964
–	Length of pedipalp femur more than half width of prosoma; femora of legs entirely smooth	*Pantopsalis snaresensis* Forster 1964

## Supplementary Material

XML Treatment for Mangatangi

XML Treatment for Mangatangi parvum

XML Treatment for Forsteropsalis pureora

XML Treatment for Pantopsalis listeri

XML Treatment for Pantopsalis cheliferoides
