# Corrigenda: Taylor CK (2013) Further notes on New Zealand Enantiobuninae (Opiliones, Neopilionidae), with the description of a new genus and two new species. ZooKeys 263: 59–73

**DOI:** 10.3897/zookeys.273.4857

**Published:** 2013-02-28

**Authors:** Christopher K. Taylor

**Affiliations:** 1 Dept of Environment and Agriculture, Curtin University, GPO Box U1987, Perth, WA 6845, Australia

The International Code of Zoological Nomenclature (ICZN 1999) requires that, for all newly published species, the type depository be clearly indicated. This was not done for *Mangatangi parvum* Taylor 2013 and *Forsteropsalis pureora* Taylor 2013. The holotypes of both species (specimen details given in the original publication) are from Te Papa Tongarewa, Wellington, New Zealand (MONZ).

The latter species was inadvertently spelt ‘*Forsteropsalis pureroa*’ at two places in the original publication. The correct spelling is *Forsteropsalis pureora*, as used elsewhere and in accordance with the cited type locality of Pureora.

I would like to thank Stephen Thorpe (Auckland University, New Zealand) for bringing these errors to my attention.
